# Analysis of a syndromic surveillance system for infectious diseases of the 31st summer World University Games in Chengdu, China

**DOI:** 10.3389/fpubh.2025.1510057

**Published:** 2025-07-11

**Authors:** Yao Wang, Yingxue Dai, Hui Liu, Songqi Feng, Yuezhu Chen, Lu Long, Ke Yan, Xunbo Du, Changhui Du, Liang Wang

**Affiliations:** ^1^Department of Infectious Disease Control and Prevention, Chengdu Center for Disease Control and Prevention, Chengdu, China; ^2^Department of Environmental and School Health, Chengdu Center for Disease Control and Prevention, Chengdu, China

**Keywords:** syndromic surveillance, mass-gathering events, public health, World University Games, Universiade

## Abstract

**Introduction:**

Chengdu hosted the 31st Summer World University Games in 2023. The movement and gathering of attendees from other cities and countries posed challenges to local public health, including an increased risk of infectious diseases or outbreaks. To manage these risks, a syndromic surveillance system targeting infectious diseases was established. This system aimed to identify suspicious cases and outbreaks early, enabling timely measures and minimizing their impact. This article reviews the surveillance mechanism, analyzes the surveillance data, and evaluates the system’s effectiveness.

**Methods:**

The mechanism of the syndromic surveillance system was described, and surveillance data were collected to evaluate strategies for infectious disease prevention and control during the Universiade. Epidemiological distributions of diseases were described, and differences were tested using chi-squared tests.

**Results:**

Surveillance was carried out among athletes, coaches, entourages, and staff from 1 week before the competition to 1 week after. The surveillance focused on symptoms such as fever, cough, diarrhea, vomiting, rash, jaundice, conjunctival redness, and sore throat, supported by a self-developed *Cloud Health Information System for Health Care Support* and *Syndromic Surveillance System for Infectious Disease*. A total of 351 subjects with disease-related symptoms, mainly including sore throat, cough, and fever, were reported. A total of 33 events of clustered symptoms were detected, none of which met the criteria for an outbreak. Fifty cases were confirmed with infectious diseases, comprising 43 cases of COVID-19, 3 cases of influenza, and 1 case each of infectious mononucleosis, chikungunya, malaria, and another infectious diarrhea.

**Discussion:**

Throughout the event, reports of disease-related symptoms were sporadically distributed, with no secondary cases or outbreaks detected and no health-related risks arising that would impact the continuation of the event. The syndromic surveillance system was sensitive and effective for the early detection and control of infectious disease outbreaks and could be implemented in other mass-gathering events.

## 1 Introduction

The Summer World University Games is the largest international sporting event for university students. The 31st edition of the Games (referred to as the Chengdu Universiade) was held in Chengdu, China, from 28 July to 8 August 2023. It attracted 6,500 participants from 113 countries (1). The Chengdu Universiade was the largest international event held in China, in terms of the number of participating countries and entrants, since the Beijing Winter Olympics and Winter Paralympics. Furthermore, it marks the first comprehensive international sports event held in the western region of China. During the event, the increased crowd density and the social and political implications of the Games often pose significant challenges and pressures on local public health (2, 3). To detect, diagnose, and control infectious diseases at an early stage, a syndromic surveillance system was established. This system was operational from 1 week prior to the competition until 1 week after its conclusion. Reports would be generated if confirmed infectious diseases or related symptoms, such as fever, diarrhea, or rash, were identified. Alerts would be activated if specific risk criteria were met, prompting health personnel to be dispatched to the field to verify and manage any suspicious risks.

## 2 Materials and methods

### 2.1 Research methods

This study systematically reviewed the syndromic surveillance system for infectious diseases, collecting surveillance data to review and assess strategies for the prevention and control of infectious diseases during the Universiade.

### 2.2 Statistical analysis

Excel 2019 was utilized to create a database for data management, while SPSS 19.0 was used for statistical analysis. The daily reported surveillance data were described using epidemiological distributions, and differences were tested using the chi-squared test.

## 3 Results

### 3.1 Mechanism of surveillance

Based on the city’s surveillance systems for legally notifiable infectious disease cases, fever and diarrhea symptoms, concurrent influenza and COVID-19, multiple respiratory pathogens, viral diarrhea, and risk factors for diseases such as cholera, dengue fever, brucellosis, and monkeypox, a symptom—based surveillance system tailored to the specific population of the Chengdu Universiade was constructed. In addition, information-based monitoring methods were used to automatically acquire and collect data.

#### 3.1.1 Objects

The subjects of surveillance include athletes, coaches, delegation entourages, international and domestic dignitaries, guests, media representatives, sponsors, council members, and staff associated with the Universiade.

#### 3.1.2 Surveillance period

The surveillance period spans from 20 July 2023 to 15 August 2023.

#### 3.1.3 Surveillance contents

Eight disease-related symptoms were monitored: fever, cough, diarrhea, vomiting, rash, jaundice, conjunctival redness, and sore throat. Along with pertinent details including their gender, age, nationality, personnel category, time of incidence, time of consultation, main symptoms, and contact information, the personal identification information of the case was hidden in the system.

### 3.2 Report criteria

#### 3.2.1 Report criteria of symptoms

A set of criteria has been established to standardize the reporting of suspicious cases, balancing the sensitivity and specificity necessary for detecting potential infectious diseases. The detailed criteria are presented in Table 1.

**Table 1:** Criteria of reporting symptoms relating to suspicious infectious diseases.

Symptoms	Report criteria
Fever	Axillary temperature over 37.3°C
Cough	Any non-physiological cough
Diarrhea	Over 3 times per day with changes of fecal trait
Vomiting	Over 2 times per day
Rash	Patches, pimples, blisters anywhere on the body
Jaundice	Yellow skin, mucous membranes or sclera due to elevated serum bilirubin
Conjunctival redness	Redness, swelling of blood vessels within the eyelids or on the eyeballs in one or both eyes
Sore throat	Any pain in throat

#### 3.2.2 Reporting criteria for infectious diseases

The reporting criteria include any suspected or confirmed cases of the following diseases: plague, cholera, severe acute respiratory syndrome (SARS), inhalational anthrax, coronavirus disease 2019 (COVID-19), monkeypox, malaria, dengue fever, Middle East respiratory syndrome (MERS), Ebola virus disease (EVD), Lassa fever, chikungunya fever, and other infectious diseases identified as requiring priority attention.

#### 3.2.3 Report criteria of clustered symptoms

An event of clustered symptoms will be identified if two individuals present with the same symptoms (e.g., fever and diarrhea) within 1 day, or if five individuals present with the same symptoms within 3 days, in the same delegation, working group, or other epidemiologically associated population, without a clear diagnosis.

### 3.3 Surveillance strategy

#### 3.3.1 Passive report from healthcare institution

Medical personnel from the *Universiade Village Health Center*, venue or hotel healthcare working group, and designated medical institution were required to input information into the *Cloud Health Information System for Health Care Support* when individuals exhibited symptoms such as fever, cough, or other disease-related symptoms. The *Syndromic Surveillance System for Infectious Disease* could then synchronize this de-identified information in real-time for further analysis.

#### 3.3.2 Proactive report from the related delegation or other units

When delegations, staff of the Universiade, or other organizational and management units identified individuals displaying symptoms related to infectious diseases, the involved parties were obligated to report the pertinent information to the designated health staff. This staff member would subsequently enter the information into the *Syndromic Surveillance System for Infectious Disease*.

#### 3.3.3 Horizontal report from customs

Personnel associated with the Universiade who exhibited abnormal symptoms during entry screening would be reported by Chengdu Customs. The on-site designated health staff would then relay the relevant information to the *Syndromic Surveillance System for Infectious Disease.*

#### 3.3.4 Action and feedback

All reports of disease-related symptoms necessitated a precise diagnosis to determine whether the subjects were associated with infectious diseases. Additionally, all reports of clustered symptom events required field investigations to ascertain the existence of infectious disease epidemics. Various laboratory tests, including those for COVID-19, influenza, norovirus, and multi-pathogen screening, were performed with informed consent to confirm the presence of pathogens.

#### 3.3.5 Quality control

All reporting was conducted by trained and qualified public health or clinical personnel from each surveillance unit. Chengdu and its district CDCs established designated working groups to verify and process warning signals in the surveillance system every 2 h. Analysis of surveillance data was conducted after 24:00, once the daily data had been locked and stabilized.

### 3.4 Analysis of surveillance

#### 3.4.1 Overview of surveillance data

A total of 6,500 athletes from 113 countries and regions across Asia, Europe, Africa, the Americas, and Oceania participated in the event. Among them were 3,512 male athletes and 2,988 female athletes. In addition, more than 20,000 technical officials and members of the operations team were involved in supporting the event (1).

Three hundred fifty-one subjects of disease-related symptoms were reported through all surveillance routes, of whom 321 subjects were reported passively by healthcare institutions (91.5%), 28 cases were reported proactively by related delegations or other units (8.0%), and 2 cases were reported laterally by Customs (0.5%).

#### 3.4.2 Disease-related symptoms

**Figure 1:**
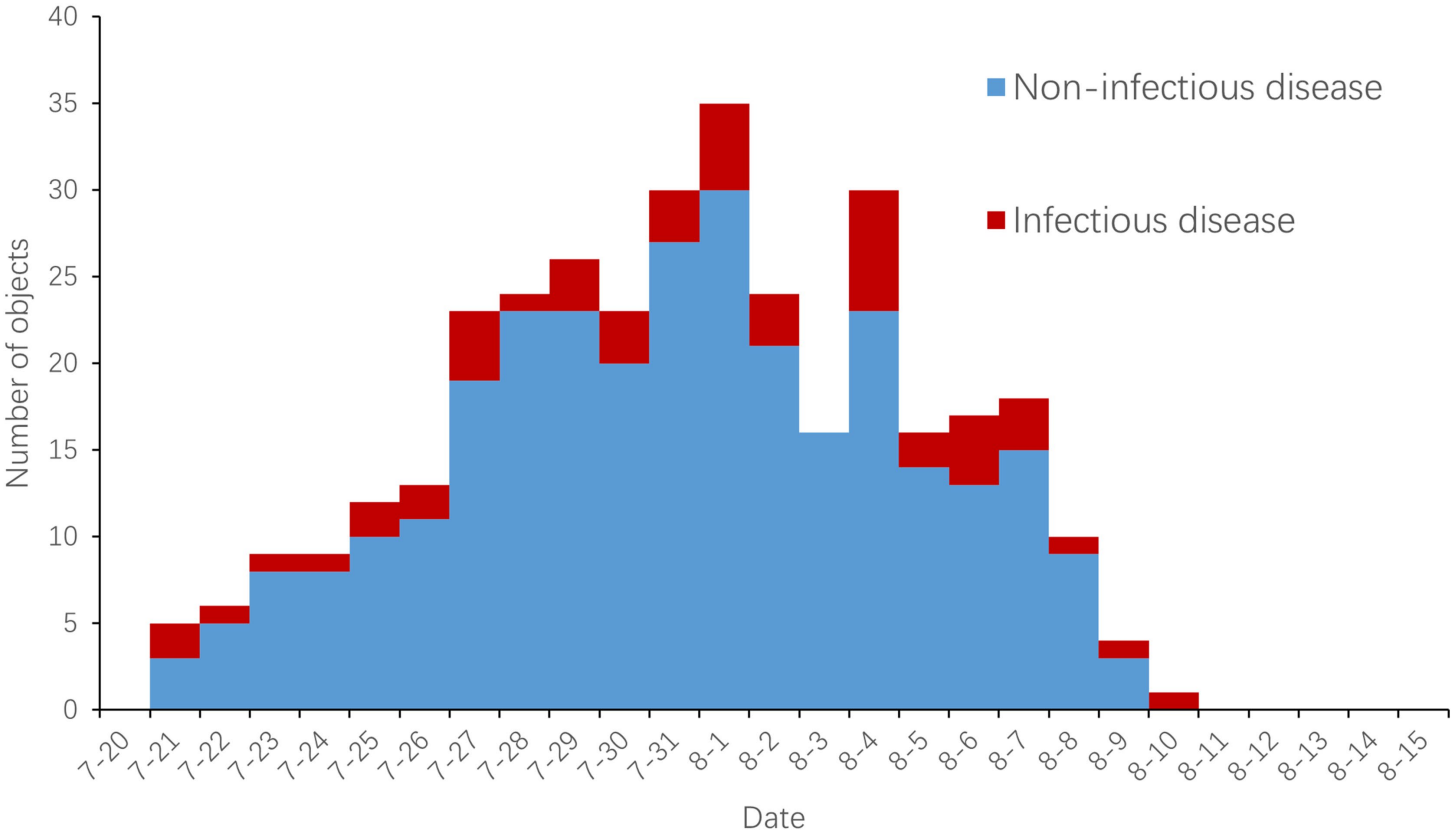
Daily distribution of disease-related symptoms and confirmed infectious diseases.

During the surveillance period, a total of 351 subjects with 481 instances of disease-related symptoms were reported, of which 109 subjects (31.1%) exhibited 2 or more disease-related symptoms. Except for jaundice, all other disease-related symptoms were reported, with sore throat, cough, fever, and diarrhea being the most common (Table 2). Of the 351 subjects, 196 (55.8%) were male and 155 (44.2%) were female; ages ranged from 7 to 77 years, with a major concentration in the 21- to 30-year-old group; 27 countries or regions (23.9%) and 41 venues or hotels (56.9%) were involved. Reports were distributed over the period from 21st July to 10th August, with a peak of 35 subjects reported on 1st August (Figure 1).

**Table 2:** Distribution of disease-related symptoms.

Disease-related symptoms	Reported times (N)	Proportion (%)
Sore throat	137	28.5
Cough	107	22.2
Fever	102	21.2
Diarrhea	91	18.9
Rash	24	5
Vomiting	15	3.1
Conjunctival redness	5	1
Jaundice	0	0
Total	481	100

#### 3.4.3 Confirmed infectious diseases

A total of 50 cases of infectious diseases were confirmed, accounting for 14.2% of cases with disease-related symptoms. Of the 50 cases, 43 were COVID-19, 3 were influenza, and there was 1 case each of infectious mononucleosis, chikungunya, malaria, and other infectious diarrhea. Cases of infectious diseases were reported daily from 21st July 21 to 10th August, except on 3rd August (Figure 1).

The proportion of subjects with fever and sore throat diagnosed as infectious diseases was higher than those without (37.3% versus 4.8%; 20.4% versus 10.3%), while the proportion of subjects with diarrhea diagnosed as infectious diseases was lower than those without (2.2% versus 18.5%). All the differences above were statistically significant (Table 3).

**Table 3:** Comparison of infectious diseases between different disease-related symptoms.

Disease-related symptoms	Proportion of infectious disease (%)	*χ^2^*	*P*
Fever			
Yes	37.3	59.691	<0.001
No	4.8		
Sore throat			
Yes	20.4	6.248	0.012
No	10.3		
Cough			
Yes	16.8	0.561	0.454
No	13.1		
Diarrhea			
Yes	2.2	13.295	<0.001
No	18.5		
Rash			
Yes	4.2	1.348	0.246
No	15		
Vomiting			
Yes	0	1.527	0.217
No	14.9		
Conjunctival redness*			
Yes	0	0.462	1
No	14.5		

*Fisher’s exact test.

#### 3.4.4 Events of clustered symptoms

According to the criteria for clustered symptoms, 33 suspected epidemics were identified. Each event was verified through epidemiological investigation, and none ultimately met the criteria for infectious disease outbreaks.

## 4 Discussion

Syndromic surveillance refers to the systematic and continuous collection and analysis of outbreak-related information prior to confirmed clinical diagnosis, enabling an early public health response (4). Compared to traditional surveillance methods that rely on clinically confirmed diagnostic data, syndromic surveillance can detect the occurrence of infectious diseases in a more timely and sensitive manner (5). Many large-scale events, such as the Sydney Olympics in 2000, the Salt Lake City Winter Olympics in 2002, and the G8 Scotland Summit in 2005, have established syndromic surveillance systems (6–9). Similar efforts have also been implemented in China during several large-scale activities in recent years (10–12).

The Chengdu Universiade was held in summer. According to the infectious disease surveillance data and the results of risk assessment, infectious diseases in the whole city mainly include COVID-19 infections, norovirus enteritis, and other common infectious diseases. At the same time, it is necessary to pay special attention to the imported risks of COVID-19 infection, influenza, monkeypox, malaria, dengue fever, etc. A total of 351 subjects of disease-related symptoms were reported during the Chengdu Universiade, of which 31.1% exhibited two or more symptoms; 50 cases of infectious diseases were diagnosed, involving COVID-19, influenza, chikungunya fever, and malaria. Compared to the syndromic surveillance results from large-scale domestic or international events, our mechanism, which focused on infectious diseases, functioned sensitively and effectively, with the number of reported cases of disease-related symptoms and confirmed infectious diseases being high (11–13). According to the surveillance and risk assessment, we put in place regular prevention and control measures such as ventilation, disinfection, and the control of mosquito density. Reports of disease-related symptoms were sporadically distributed, with no secondary cases or outbreaks detected. Additionally, no health-related risks arose that would impact on the event’s continuation. Chengdu had not reported local cases of chikungunya fever and malaria in recent years, but both cases were successfully identified and effectively controlled with the help of this surveillance, resulting in no subsequent cases and no additional infectious disease burden added to the city, demonstrating the value of the surveillance.

Among the various symptoms, cough, sore throat, fever, and diarrhea accounted for the highest proportion, while jaundice was not reported, which was consistent with the Beijing Olympic Games (14) and the Guangzhou Asian Games (15). Only one case of jaundice was reported in the Shenzhen Universiade (11). Among the various symptoms monitored, fever proved to be more significant for the early identification of infectious diseases. This may be due to the fact that respiratory and insect-borne diseases often present with more identifiable symptoms, such as fever. These findings suggest that fever should be given greater emphasis in future syndromic surveillance. Diarrhea, on the other hand, was more often observed in non-infectious disease cases and may be associated with other non-infectious intestinal diseases, requiring attention to differential diagnosis.

This surveillance also found that healthcare institutions set up at Universiade Village, venues, and hotels contributed most of the surveillance data, while Customs screening and proactive reporting supplemented this, suggesting that the comprehensive construction of an input-side, city-side, event-side syndromic surveillance system for infectious diseases was sensitive and effective at large-scale events. The establishment of an intelligent surveillance system, along with the online and offline cooperation of CDCs and medical institutions at all levels, was key to the successful completion of disease surveillance and epidemic management. Compared to the syndromic surveillance systems of large-scale events both domestically and internationally, our system was sensitive and effective, demonstrating remarkable results in disease prevention and control, which can be widely implemented in future large-scale events for disease control and prevention.

## Data Availability

The original contributions presented in the study are included in the article/supplementary material, further inquiries can be directed to the corresponding author.
